# Seroepidemiology of *Plasmodium* species infections in Zimbabwean population

**DOI:** 10.1186/s12936-016-1325-3

**Published:** 2016-05-10

**Authors:** Seth A. Amanfo, Takafira Mduluza, Nicholas Midzi, David R. Cavanagh, Francisca Mutapi

**Affiliations:** Institute of Immunology & Infection Research and Centre for Immunity, Infection & Evolution, Ashworth Laboratories, School of Biological Sciences, University of Edinburgh, King’s Buildings, Charlotte Auerbach Rd, Edinburgh, EH9 3FL UK; Biochemistry Department, University of Zimbabwe, P.O. Box MP167, Mount Pleasant, Harare, Zimbabwe; School of Laboratory Medicine and Medical Sciences, University of KwaZulu Natal, Durban, South Africa; College of Health Sciences, Department of Medical, Microbiology, University of Zimbabwe, P.O. Box A178, Avondale, Harare, Zimbabwe

**Keywords:** Antibody, Merozoite surface protein 1 (MSP-1_19_), *Plasmodium*, Microscopy

## Abstract

**Background:**

Individuals living in malaria-endemic regions may be exposed to more than one *Plasmodium* species; there is paucity of data on the distribution of the different species of *Plasmodium* in affected populations, in part due to the diagnostic method of microscopy, which cannot easily differentiate between the species. Sero-epidemiological data can overcome some of the shortcomings of microscopy.

**Methods:**

The specificity of IgG antibodies to recombinant merozoite surface protein 1 (MSP-1_19_) derived from four human *Plasmodium* species (*Plasmodiumfalciparum*, *Plasmodiumvivax*, *Plasmodiummalariae*, *Plasmodiumovale*) was investigated using competition enzyme-linked immunosorbent assay. Subsequently, these antigens were used to determine the exposure prevalence to the different *Plasmodium* species in serum samples of participants. One-hundred individuals, aged five-18 years, from each of the three *Plasmodium* meso-endemic Zimbabwean villages (Burma Valley, Mutoko, Chiredzi) were recruited in the study.

**Results:**

The study demonstrated that the host serum reactivity to MSP-1_19_ antigens was species-specific and that no cross-reactivity occurred. The overall prevalence of antibody response to MSP-1_19_ antigens was 61 % in Burma Valley, 31 % in Mutoko and 32 % in Chiredzi. Single species IgG responses to MSP-1_19_ were most frequent against *P*. *falciparum,* followed by *P*. *malariae* and *P*. *ovale*, with responses to *P*. *vivax* being the least prevalent. Interestingly, 78–87 and 50 % of sera with IgG responses to *P*. *malariae* and *P*. *ovale* MSP-1_19_, respectively, also had IgG specific response for *P*. *falciparum* MSP-1_19_ antigens, indicating that exposure to these species is a common occurrence in these populations. Single species IgG responses to the non-falciparum species were at a very low frequency, ranging between 0 and 13 % for *P*. *malariae*.

**Conclusions:**

There is evidence of a higher exposure to the non-falciparum parasite species than previously reported in Zimbabwe. The recombinant MSP-1_19_ antigens could be used as additional diagnostic tools in antibody assays for the detection of exposure to the different *Plasmodium* species. The results also introduce an interesting concept of the co-infection of non-falciparum *Plasmodium* almost always with *P. falciparum*, which requires further validation and mechanistic studies.

**Electronic supplementary material:**

The online version of this article (doi:10.1186/s12936-016-1325-3) contains supplementary material, which is available to authorized users.

## Background

Malaria is a major public health problem in sub-Saharan Africa, and is responsible for over half a million deaths annually, especially in children under the age of 5 years [1]. Four major species of the protozoan parasite, *Plasmodium*, (*Plasmodiumfalciparum*, *Plasmodium vivax*, *Plasmodium malariae*, *Plasmodium ovale*) cause human malaria in sub-Saharan Africa. In malaria-endemic countries, there is an overlap in the geographic distribution of the different *Plasmodium* species and the *Anopheles* mosquito vectors that transmit these parasites, and that individuals may be exposed to, and harbour multiple *Plasmodium* species [2]. However, the epidemiology of the different *Plasmodium* species in endemic human populations is not well documented [3]. Diagnosis of malaria in endemic clinical settings is predominantly by the ‘gold standard’ blood film microscopic examination, and rapid diagnostic tests (RDT), both of which lack sensitivity in differentiating the species of *Plasmodium* causing malaria. Microscopic examination has several limitations such as the inability to detect low levels of parasitaemia, and the difficulty in species differentiation owing to subtle differences in the morphology of blood stage parasites [4]. This results in the species of *Plasmodium* causing disease being rarely reported, and almost all cases of malaria are therefore attributed to *P*. *falciparum*, the species causing the most serious form of malaria [3]. This has led to underestimates of the prevalence of both mixed-species and non-falciparum species infections [5]. These non-falciparum species are of significant clinical importance; for example, *P*. *vivax* and *P*. *ovale* which form latent liver stage ‘hypnozoites’ are capable of causing disease several months or years after the primary infection [6]. Incidences of the diagnosis of systemic diseases caused by *P*. *malariae* several months or years after people have returned from malaria-endemic regions have been reported [7]. In some cases, drug treatment failure attributable to the misdiagnosis of primary infections caused by the non-falciparum species or as co-infecting species with *P*. *falciparum* have been observed [8]. While PCR typing of infecting *Plasmodium* species is not frequently available or applicable in many African field settings, most RDTs may not differentiate non-falciparum species [3]. There is an urgent need for additional diagnostic tools [2–4] capable of rapid detection of all four infecting *Plasmodium* species for effective treatment and control of malaria.

In this study, a new assay has been developed that detects exposure to all four human *Plasmodium* species based on serum antibody responses to merozoite surface protein 1 (MSP-1). The surface of the invasive merozoite is coated in MSP-1 that constitutes 31 % of the GPI-anchored proteins on *P. falciparum* merozoites [9]. MSP-1 is expressed by all four human *Plasmodium* species. In *P. falciparum*, MSP-1 undergoes two proteolytic cleavages resulting in a C-terminal MSP-1_19_ fragment that is carried into the erythrocyte during merozoite invasion [10, 11]. Until recently, only the MSP-1 genes of *P*. *falciparum* and *P*. *vivax* had been characterized. Recently, the sequences of the MSP-1_19_ gene fragments for *P*. *malariae* and *P*. *ovale* have been determined with limited characterization of the responses to these parasite proteins [12]. Although the gene sequences of MSP-1_19_ antigens are unique to each of these four *Plasmodium* species, extensive homology can be found among them. The number and relative positions of cysteine residues within the C-terminus fragments of MSP-1_19_ are comparable in all four *Plasmodium* species [12]. For example, there are about 32 amino acid sites within the MSP-1_19_ gene where all four parasite species share the same amino acid, and about 30 sites where the same amino acid is conserved in two or three species (Additional file 1: Figure S1). To date, there has been no field study using MSP-1_19_ antigens from all four malaria parasite species to characterize the epidemiology of exposure to *Plasmodium* in any African population.

In Zimbabwe over half of the population are exposed to malaria, with *P*. *falciparum* being the predominant species, accounting for almost all cases of the disease [13]. There is little epidemiological data of exposure to non-falciparum species and/or mixed *Plasmodium* infections in Zimbabwe. The aim of this study is to determine the species specificity of IgG antibody responses to recombinant *Plasmodium* MSP-1_19_ antigens in three meso-endemic villages of Zimbabwe: Burma Valley, Mutoko and Chiredzi. Using these antigens as diagnostic tools, this study describes the sero-epidemiology of multiple *Plasmodium* species infections in these study sites.

## Methods

### Study sites and population

Serum samples were collected in three Zimbabwean villages where malaria parasite transmission is described as meso-endemic [13], as part of studies investigating the immuno-epidemiology of schistosomiasis in villages with *Plasmodium* co-infection. The study sites were Burma Valley in the northeast, where samples were collected in 1994, Chiredzi in the southeast where samples were collected in 1999 and Mutoko in central Zimbabwe, where samples were collected in 2003. The study cohort consisted of 100 participants aged between 5 and 18 years (both males and females) in each study site (Table 1). Antibody responses to merozoite surface proteins were detected using enzyme-linked immunosorbent assays (ELISA).

**Table 1:** Summary of study population

Study area	Age range (years)	Median age (years)	Sex	
			Male (%)	Female (%)
Burma valley	6–15	10	49	51
Mutoko	5–18	9.5	36	64
Chiredzi	7–16	11	52	48

100 individuals from each village were recruited into the study

### Ethical approval and consent

The studies in the different study sites received ethical approval from the Medical Research Council of Zimbabwe. Permission to conduct the work in each of the three villages was obtained from the Provincial Medical Director, the District Educational Officer and Heads of schools in the study sites. Project aims and procedures were fully explained to the study participants and/or their guardian. Informed oral consent/assent was obtained from parents/guardians, or participants if older than 10 years, prior to enrolment of the participants into the study. The participants were recruited into the study on a voluntary basis and were free to withdraw with no further obligation.

### Recombinant antigens

MSP-1 antigens used in the ELISAs were expressed in *Escherichiacoli* transformed with pGEX-derived plasmid constructs [14–17] as recombinant proteins fused to glutathione S-transferase (GST). These were purified by affinity chromatography using HiTrap glutathione Sepharose columns on an AKTAprime system and quantified by the Bradford protein assay.

### Serology

Sera were tested by ELISA for the presence of IgG antibodies able to recognize the recombinant merozoite surface proteins as an indication of recent or current exposure; 96-well plates (Immulon4 HBX; Dynex, Greiner Microlon) were coated with 100 μL of 0.5 μg/mL of recombinant antigen in carbonate bicarbonate buffer (15 mM Na_2_CO_3_, 35 mM NaHCO_3_, pH 9.4) and incubated overnight at 4 °C in a humidified atmosphere. Plates were washed four times in washing buffer (0.05 % Tween-20 in PBS) using Skatron Skanwasher to remove unbound antigens and blotted on paper towels (Kimberley Clark 3-ply hand towels Cat No. 6771). Free binding sites in wells were blocked with 200 μL per well of blocking buffer (1 % (w/v) skimmed milk powder in the PBS buffer) for 5 h at room temperature and then plates further washed four times. Human serum diluted 1:500 in the blocking buffer (100 μL per well) was added in duplicate to the Ag-coated wells and incubated overnight at 4 °C. After four washes, the wells were incubated for 3 h at room temperature with 100 μL per well of horseradish peroxidase-conjugated rabbit antihuman IgG (1:5000) (Dako Ltd, High Wycombe, UK). Plates were washed four times to remove unbound secondary antibody before reaction development with 100 μL of substrate buffer [(0.04 mg/mL of *o*-phenylenediamine; Sigma, St Louis, MO, USA; 0.012 % H_2_O_2_ in development buffer (24.5 mM citric acid monohydrate and 52 mM Na_2_HPO4, pH 5.0)] for 10–15 min at room temperature. An unstopped positive control plate was read at an optical density (OD) 450 nm, with an OD 450 nm of 0.7–0.8 taken to be equivalent to OD 492 nm of 2.5–3.0. The reaction was stopped by the addition of 25 μL of 2 M H_2_SO_4_ per well, and OD was measured at 492 nm (Labsystems Multiskan Ascent microtitre plate reader). GST protein, purified from *Escherichia coli* transfected with pGEX-2T alone, was used as a control to determine the non-specific (background) binding of human IgG to the GST. Corrected OD values for each plasma sample were calculated by subtracting the mean OD value of wells containing control GST protein from the mean OD value obtained with each test MSP-1 antigen. Cut-off values at which binding of Ab from malaria-exposed individuals was regarded as significantly above background were calculated as corrected OD above the mean plus 4 standard deviation of OD readings obtained with sera from eight Scottish blood donors with no history of exposure to malaria. The same positive controls (pooled sera from Brefet, The Gambia) were run in duplicate, on each plate, to allow for standardization of plate-to-plate variations.

### Competition ELISA

Competition ELISA was performed for individuals with substantial antibody reactivity to more than one MSP-1_19_ antigen, to assess whether human anti-MSP-1_19_ IgG antibodies specific for MSP-1_19_ were species-specific or cross-reacted with each other. Serum was pre-incubated with different concentrations of MSP-1_19_ antigen, and then added to the wells of microtitre plates coated with either the homologous or heterologous MSP-1_19_ antigen. The rationale of the competition ELISA is that appropriate antigen epitopes will react with their corresponding paratopes in the sera, so that with increasing antigen concentration, all paratopes in the sera react with the antigen, leaving none available to bind to antigen on the plate [18]. In the case of antigens without corresponding paratopes in the sera, there will be no prior reactivity between the serum and antigens, regardless of the antigen concentration. The same ELISA protocol above was followed with slight modification. Plates were coated with recombinant MSP-1_19_ Ag and incubated overnight at 4 °C. Serum was diluted (1:500) and pre-incubated with increasing concentrations (0–10 μg/mL) of soluble competing homologous or heterologous Ag, i.e., with up to 20-fold excess over the 0.50 μg/mL immobilized Ag to allow sera to bind to the antigen before reacting with the antigen bound on the plate, then tested on the plate-bound Ag overnight. This was followed by washing and incubation with a horseradish peroxidase-conjugated second Ab, as described above.

### Statistical analyses

To determine if exposure prevalence derived from single species data differed from that based on multiple species, Chi square (χ^2^) tests were used.

## Results

### Specificity of *Plasmodium* MSP-1_19_ antigens

**Fig. 1:**
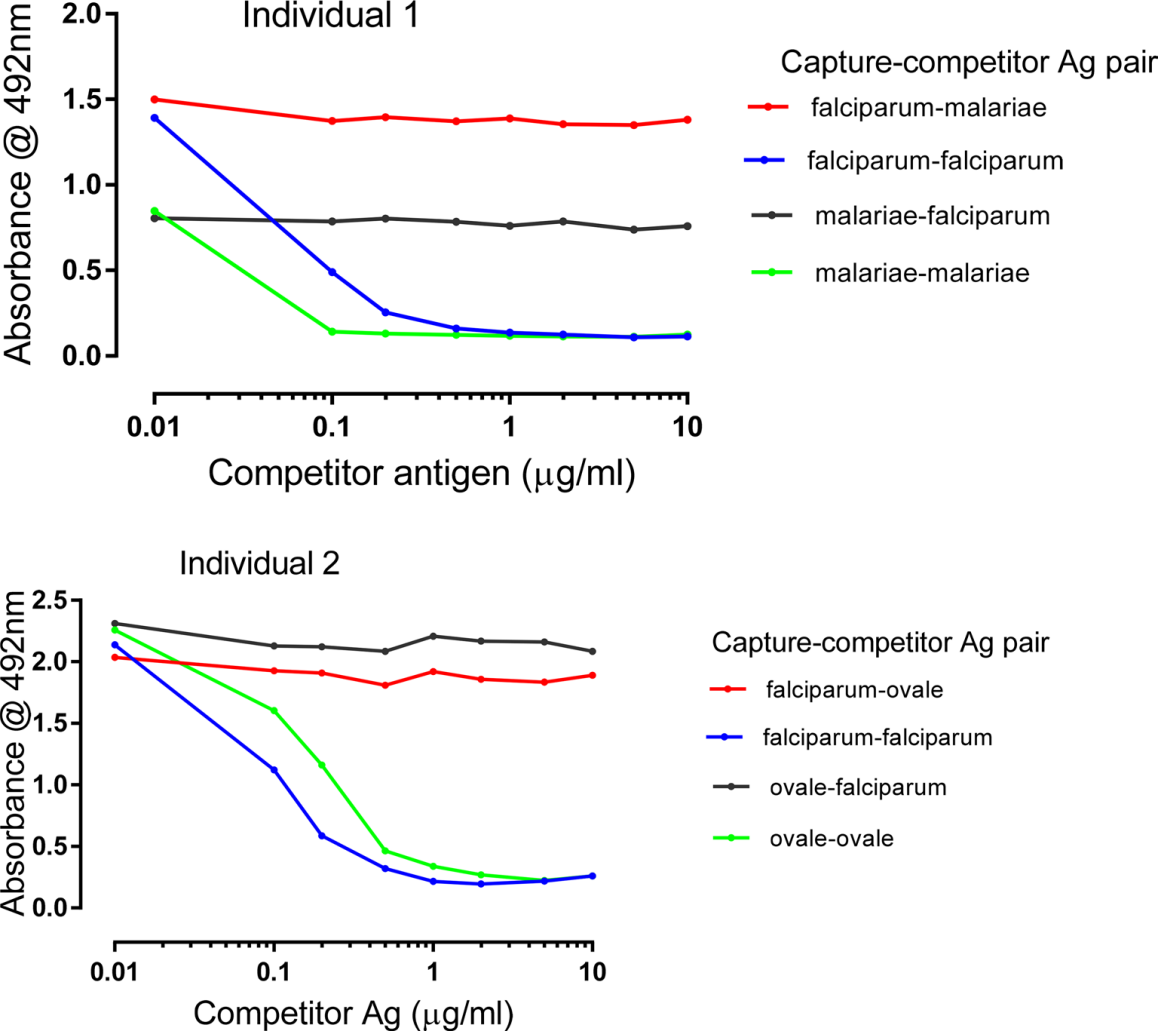
Competition ELISA showing species specificity of Abs to recombinant *Plasmodium* MSP-1_19_ antigens for individuals 1 and 2. Sera were tested at 1:500 dilution.* Legends* indicate the pairs of competing antigens used, with the well-bound capture antigen listed first and the competing homologous or heterologous antigen second. The capture antigens were coated at 50 ng/well. The *x-axis* indicates increasing concentrations of competing antigen added to the diluted sera

Competition ELISA showed that in sera reactive against recombinant MSP-1_19_ antigens from more than one parasite species, anti-MSP-1_19_ IgG antibody responses were species-specific and did not cross-react. As a positive control, when *P*. *falciparum* MSP-1_19_ antigen was coated onto microtitre plates (as capture Ag), sera pre-incubated with *P*. *falciparum* MSP-1_19_ (competing homologous Ag), were inhibited from binding in a dose-dependent manner (Fig. 1). This inhibition occurred at competing homologous Ag concentration as low as 0.1 μg/mL. Similar results were observed when the homologous competitor Ags were either *P*. *malariae* or *P*. *ovale* MSP-1_19_. To assess anti-MSP-1_19_ cross-reactivity, *P*. *falciparum* MSP-1_19_ antigen was coated onto microtitre plates and dual specificity sera were pre-incubated with increasing concentrations of the heterologous *P*. *malariae* or *P*. *ovale* MSP-1_19_ antigens. IgG binding to *P*. *falciparum* MSP-1_19_ antigen was not inhibited by soluble heterologous *P*. *malariae* or *P*. *ovale* MSP-1_19_, even at concentration 20 times the capture antigen. This was also true when the coating and competing antigens were reversed in the assay (Fig. 1).

### Prevalence of human IgG antibodies to recombinant MSP-1_19_ antigens from four *Plasmodium* species

Antibody recognition of the panel of four *Plasmodium* recombinant MSP-1_19_ antigens was tested by ELISA against 100 sera from each of the three study sites (Burma Valley, Mutoko, Chiredzi). The observed overall prevalence of IgG response to all recombinant MSP-1_19_ antigens was 61, 31 and 32 % in the Burma Valley, Mutoko and Chiredzi villages, respectively (Table 2). There were no significant differences between the exposure prevalence between the villages (Burma Valley vs Mutoko: χ^2^ = 0.002, df = 1, *P* = 0.97), (Burma Valley vs Chiredzi: χ^2^ = 0.423, df = 1, *P* = 0.52) and (Mutoko vs Chiredzi: χ^2^ = 0.001, df = 1, *P* = 0.97).

**Table 2:** Prevalence of antibody reactivity to single and multiple *Plasmodium* spp. anti-MSP-1_19_

Study area	Overall prevalence (%)	Single *Plasmodium* spp. response	Multiple *Plasmodium* spp. response	Single spp. anti-MSP-1_19_ Ab responses			Multiple spp. anti- MSP-1_19_ Ab responses				
				Falciparum	Malariae	Ovale	Falciparum/malariae only	Falciparum/ ovale only	Falciparum/ vivax only	Malariae/ ovale only	Falciparum/ malariae and ovale
Burma valley (n = 100)	61	30 (49.2)	31 (50.8)	24 (80)	3 (10)	3 (10)	26 (83.9)	2 (6.5)	0	1 (3.1)	2 (6.5)
Mutoko (n = 100)	31	15 (48.4)	16 (51.6)	11 (68.8)	0	4 (31.2)	6 (37.5)	2 (12.5)	0	3 (18.8)	5 (31.2)
Chiredzi (n = 100)	32	23 (71.9)	9 (28.1)	18 (78.3)	3 (13)	2 (8.7)	6 (66.7)	0	1 (11.1)	0	2 (22.2)

Values indicate the number of responders while those in parenthesis are expressed as % of responders in the respective categories

*n* number of individuals

### Single *vs* multiple *Plasmodium* species anti-MSP-1_19_ responses

Of the individuals with anti-MSP-1_19_ responses in all three study areas, Burma Valley (n = 61), Mutoko (n = 31) and Chiredzi (n = 32), single species responses were the most common occurrence, and these were predominantly directed against *P*. *falciparum* MSP-1_19_ antigens (80, 68.8 and 78.3 %, respectively), with responses to *P*. *malariae* or *P*. *ovale* ranging from 0 to 31.2 % (Table 2). The proportion of individuals with single *versus* multiple species anti-MSP-1_19_ responses in all three study villages were comparable.

In responders with antibodies to multiple *Plasmodium* species, antibody responses to the non-falciparum species were almost always accompanied by responses against *P*. *falciparum* MSP-1_19_ antigens in all three study areas. Multiple responses to *P*. *falciparum*- and *P*. *malariae* MSP-1_19_ were the most common, with about 78–87 and 50 % of all sera with IgG responses to *P*. *malariae* and *P*. *ovale* MSP-1_19_, respectively, also having IgG-specific response for *P*. *falciparum* MSP-1_19_ antigens. The high exposure prevalence of IgG responses to *P*. *malariae* MSP-1_19_ antigens suggests that infection with this parasite species is at a higher frequency in these populations than has been previously reported, and are predominantly co-responses to the main response to *P*. *falciparum* MSP-1_19_ antigen.

**Fig. 2:**
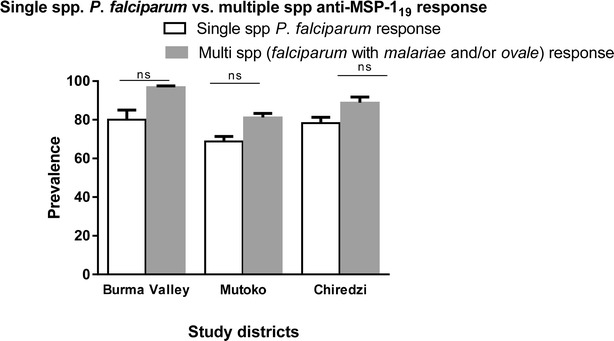
Prevalence of single *Plasmodium* *falciparum* vs multiple species anti-MSP-1_19_ responses. Observed prevalence of single spp. *P*. *falciparum* anti-MSP-1_19_ responses were compared with multiple responses involving *P*. *falciparum* with *P*. *malariae* and/or *P*. *ovale* in all three study sites

While there were no single species responders to *P*. *malariae* MSP-1_19_ in Mutoko, a much higher proportion (31.2 %) responded to three parasite antigens (*P*. *malariae*, *P*. *falciparum,P*. *ovale*) compared to the other two villages. Only one individual in Chiredzi had an antibody response to *P*. *vivax* antigen in addition to a response to *P*. *falciparum* (Table 2). When single species responses involving only *P*. *falciparum* was compared to multiple species responses involving *P*. *falciparum* with *P*. *malariae* and/or *P*. *ovale*, no significant difference was observed in all three villages (Fig. 2).

When the cohort was divided into two age groups, based on the median ages (i.e., those below 10 years and those 10 years and above), it was observed that the overall exposure prevalence in the two age groups were comparable in both Burma Valley (χ^2^ = 3.5, df = 1, *P* = 0.06) and Mutoko (χ^2^ = 0.05, df = 1, *P* = 0.83). However, in Chiredzi a significantly higher exposure prevalence was observed in responders aged 10 years and above compared to those below 10 years (χ^2^ = 6.13, df = 1, *P* = 0.01) (Table 3). Differences between the two age groups in responders to single and multiple species in all three study areas were not compared because of the smaller sample sizes involved.

**Table 3:** Prevalence of antibody reactivity to single and multiple *Plasmodium* spp. anti-MSP-1_19_ by age

Study area	Age range years (n)	Overall prevalence (%)	Single *Plasmodium* spp. response	Multiple *Plasmodium* spp. response	Single spp. anti-MSP-1_19_ Ab responses			Multiple spp. anti- MSP-1_19_ Ab responses				
					Falciparum	Malariae	Ovale	Falciparum/malariae only	Falciparum/ovale only	Falciparum/vivax only	Malariae/ovale only	Falciparum/malariae/ovale
Burma valley (n = 100)	6–9 (45)	33 (73.3)	15 (45.5)	18 (54.5)	13 (86.7)	1 (6.6)	1 (6.6)	15 (83.2)	1 (5.6)	0	1 (5.6)	1 (5.6)
	10–15 (55)	28 (50.9)	15 (53.6)	13 (46.4)	11 (73.3)	2 (13.3)	2 (13.3)	11 (84.6)	1 (7.7)	0	0	1 (7.7)
Mutoko (n = 100)	5–9 (50)	16 (32)	7 (43.8)	9 (56.2)	6 (85.7)	0	1 (14.3)	3 (33.3)	2 (22.2)	0	2 (22.2)	2 (22.2)
	10–18 (50)	15 (30)	8 (53.3)	7 (46.7)	5 (62.5)	0	3 (37.5)	3 (42.8)	0	0	1 (14.4)	3 (42.8)
Chiredzi (n = 100)	7–9 (25)	3 (12)	2 (66.7)	1 (33.3)	2 (100)	0	0	1 (100)	0	0	0	0
	10–16 (75)	29 (38.7)	21 (72.4)	8 (27.6)	16 (76.2)	3 (14.3)	2 (9.5)	5 (62.5)	0	1 (12.5)	0	2 (25)

Values indicate the number of responders while those in parenthesis are expressed as % of responders in the respective categories

*n* number of individuals

## Discussion

In this current study, IgG responses to recombinant MSP-1_19_ antigens (an indication of prior exposure to *Plasmodium* antigens) from the four major human *Plasmodium* species were evaluated in three Zimbabwean villages with meso-endemic malaria transmission dynamics. Individuals living in malaria-endemic regions may harbour multiple *Plasmodium* species owing to the geographical overlap of the four major human *Plasmodium* species [4, 19, 20]. Malaria diagnosis in most African field settings is largely by microscopy of blood films, which reports the presence or absence of *Plasmodium* parasites without cognisance to the species causing disease. Low-level parasitaemia of the non-falciparum species in mixed infection with *P*. *falciparum* accounts for the misdiagnosis of these species. There have been case study reports of treatment failures [21] and acute renal injury [22] attributable to undiagnosed *P*. *malariae* infection or co-infection. Knowledge of the type of infecting species is therefore essential for effective treatment as well as the implementation of control programmes. IgG responses to *P*. *falciparum* MSP-1_19_ antigens have been shown to rise following clinical episodes of malaria and decline in the absence of the disease [15]. In the current study, the antibody response to *Plasmodium* species recombinant MSP-1_19_ antigens in humans was seen to be highly species-specific. Furthermore, the study showed that the responses were not cross-reactive, despite the amino acid sequence similarities between the four *Plasmodium* MSP-1_19_ antigens. In experimental monkey and human studies utilizing all four *Plasmodium* species MSP-1_19_ antigens, a superior sensitivity was seen when compared to commercially available antibody assays which only utilize MSP-1_19_ antigens from *P*. *falciparum* and *P*. *vivax* and depend on cross-reactivity in detecting the other two species [23]. The specificity of antibodies to these antigens supports the evidence that these antigens could be used in pan-malaria diagnostic assay to enable the rapid detection of the type of *Plasmodium* species causing malaria [23].

In many malaria-endemic countries in sub-Saharan Africa, *P*. *falciparum* is the predominant species that causes malaria, thus it was not surprising that the antibody response to *P*. *falciparum* MSP-1_19_ antigens was predominant in all three study sites. Since *P*. *falciparum* infections have higher parasitaemias than the other malaria parasite species [20, 24], it is likely that individuals will have a stronger immune response to *P*. *falciparum* infection. The novel results from this study were the indication that the exposure prevalence of *P*. *malariae* and *P*. *ovale* is higher, as previous reports have attributed about 98 % of malaria in Zimbabwe to be caused by *P*. *falciparum*. More importantly, the observed higher exposure prevalence of *P*. *malariae* in the Burma Valley district was striking, as reports suggest that this species only accounts for between 1 and 2.6 % of all malaria cases by light microscopy [25–27].

Microscopy has long been known to underestimate the prevalence of the non-falciparum species owing to difficulties in distinguishing the subtle differences in the morphology of the different species as well as the challenge posed in detecting minority species in a blood film with high density *P*. *falciparum* parasitaemia. It is therefore not surprising that these assays detected a higher sero-prevalence of these species, as this also reflects recent and concurrent parasite exposure. Studies employing nucleic acid based techniques for *Plasmodium* parasite detection and species identification in some African countries have reported prevalence of the non-falciparum species to be between 1 and 17 % [20, 24].

While antibody responses to single species *P*. *falciparum* antigens were common, single species responses to *P*. *malariae* and *P*. *ovale* antigens were infrequently detected. A significant proportion of individuals with IgG responses to *P*. *malariae* and/or *P*. *ovale* MSP-1_19_ almost always had responses to *P*. *falciparum* MSP-1_19_. This results support the findings of a recent study in Ghana, which reported frequent detection of *P*. *malariae* and *P*. *ovale* in individuals who are also PCR positive for *P*. *falciparum* [28]. The reasons for this co-occurrence of the non-falciparum species with *P*. *falciparum* may be both epidemiological and biological. Of the epidemiological reasons, it has been suggested that the same *Anopheles* mosquito circulating in a population might be responsible for the simultaneous or sequential inoculation of the different species [4], thereby increasing the likelihood of multiple species infections. Biological reasons may include selective advantages for these minor species when co-infecting with *P*. *falciparum*. For example, due to density-dependent regulation of immune responses directed against the majority species (*P*. *falciparum*), these non-falciparum species may be able to evade host immune responses and establish disease [29, 30]. There are parallels in other infectious diseases, such as the obligate satellite virus hepatitis D, which is unable to establish disease independent of hepatitis B virus [31]. Hepatitis D virus co-infection in Hepatitis B-infected individuals worsens hepatic damage and inflammation, and is more likely to lead to hepatocellular carcinoma [32, 33]. The results show some single species *P*. *malariae* responses, indicating that this species is capable of establishing infection independent of other *Plasmodium*. However, the significant proportion of individuals with co-occurrence of antibody responses to *P*. *falciparum* suggests a possible dependency on *P*. *falciparum* receptors or proteins for successful disease by *P*. *malariae*. These non-falciparum species, which usually exist as part of a complex mixed-infections with *P*. *falciparum* [2, 34] may cause chronic, sub-clinical disease with potential health consequences, including treatment failure, disease relapse and long-term systemic consequences [5–7]. A recent study in Indonesia found *P*. *malariae* to be associated with a lower mean haemoglobin, nephrotic syndrome and death [27].

Antibody responses to *P. vivax* MSP-1_19_ were rarely observed in this study. *Plasmodium vivax* requires the Duffy antigen to establish a successful infection [35], and is predominantly endemic in Asian and Latin American countries. It has long been known that the Duffy antigen is absent in most African populations [35]; it was therefore not surprising to observe a low frequency of responses to *P*. *vivax* MSP-1_19_. In recent years however, there have been reports of *P*. *vivax* infections in both Duffy positive and negative individuals in Cameroon [36] suggesting that this species might have evolved and adapted to using other receptors to invade erythrocytes and establish disease.

Serological responses generally increase with age. In this present study, age was not a confounding factor in Burma Valley and Mutoko, while is Chiredzi responders 10 years old and above had a higher overall exposure prevalence to parasite antigens. Although all three villages are described as meso-endemic, the observed differences in age responses could be due to the respective transmission dynamics of the different seasons in which sampling was done. In very low and unstable malaria transmission areas such as Daraweesh in eastern Sudan, reports suggest that the age dynamics associated with malaria and serological responses are not apparent, as malaria affects all age groups [37, 38].

## Conclusions

This study has shown for the first time that IgG antibodies to recombinant MSP-1_19_ antigens of the four major human *Plasmodium* species are species-specific. The study also demonstrates that in Zimbabwean populations exposed to *Plasmodium* infections, the prevalence of the non-falciparum species responses is higher than previously reported in many other cross-sectional studies (reviewed in [3]). Finally, the study has demonstrated that a high proportion of individuals with antibody responses to non-falciparum MSP-1_19_ antigens also had antibodies to *P*. *falciparum* MSP-1_19_. MSP-1_19_ antigens from all four major species of human malaria parasite offer a potential diagnostic tool for the rapid detection of exposure to multiple *Plasmodium* species such as in blood transfusion screening services. It remains to be established if these exposure responses to multiple *Plasmodium* species indicated by the serological survey correspond to concurrent or sequential exposure to previous or current infections.

## Additional file

10.1186/s12936-016-1325-3 Sequence homology of *Plasmodium* MSP-1_19_ antigens. Dots or semi-colons (. or :) indicate gene site where the same amino acid is shared between two or three *Plasmodium* species, while the stars (*) indicate conserved amino acid present in all four *Plasmodium* species.

## Abbreviations

MSP: merozoite surface protein
RDT: rapid diagnostic test
PCR: polymerase chain reaction
ELISA: enzyme-linked immunosorbent assay
GST: glutathione S-transferase
OD: optical density
