# Supplementary material for: Seroepidemiology of Plasmodium species infections in Zimbabwean population
Source: Malar J. 2016 May 10;15:267. doi: 10.1186/s12936-016-1325-3 (PMC4863323; doi:10.1186/s12936-016-1325-3)
Supplement: Supplementary file 1 — 10.1186/s12936-016-1325-3 Sequence homology of Plasmodium MSP-119 antigens. Dots or semi-colons (. or :) indicate gene site where the same amino acid is shared between two or three Plasmodium species, while the stars (*) indicate conserved amino acid present in all four Plasmodium species. [file 12936_2016_1325_MOESM1_ESM.docx]

Supplementary figure S1: **Sequence homology of *Plasmodium* MSP-1_19_ antigens.**  Dots or semi-colons (. or :) indicate gene site where the same amino acid is shared between two or three *Plasmodium* species, while the stars (*) indicate conserved amino acid present in all four *Plasmodium* species.
